# Giant chondrosarcoma of rib: surgical resection and reconstruction with titanium bar, polypropylene mesh, and muscle advancement flap

**DOI:** 10.1186/1749-8090-10-S1-A133

**Published:** 2015-12-16

**Authors:** Mertay Boran, Ertay Boran, Barış Yiyit, Nisa Elif Ünlü, Birgül Onec

**Affiliations:** 1 Department of Thoracic Surgery, Duzce University Faculty of Medicine, Duzce, Turkey; 2 Duzce University Faculty of Medicine, Department of Anaesthesiology and Reanimation, Duzce, Turkey; 3 Department of Plastic and Reconstructive Surgery, Duzce University Faculty of Medicine, Duzce, Turkey; 4 Department of Radiology, Duzce University Faculty of Medicine, Duzce, Turkey; 5 Department of Haematology, Duzce University Faculty of Medicine, Duzce, Turkey

## Background/Introduction

Primary malignant tumours of the chest wall are rare. Chondrosarcoma is the most common malignancy of the sternum. Chondrosarcoma derived from rib is rare. Wide resection treatment is important because it is resistant to chemotherapy and radiotherapy.

## Aims/Objectives

We report a case of giant chondrosarcoma arising on the 6th rib of a 76-year-old man which is growing slowly for 20 years and is treated with chest wall resection and different reconstruction way.

## Method

Examination of 76-year-old male patient with anemia revealed a giant mass at anterior side of the right hemithorax. The mass with the story of slowly growing for 20 years which causing pain in the last 15 days revealed no involvement in bone scintigraphy. His chest CT showed erosion and destruction of 6.rib and 11 × 7.8 cm mass with millimetric calcifications and intermediate indistinct borders of the pectoral muscles. True-cut biopsy revealed findings consistent with chondrosarcoma.

## Results

Tumor was resected together with the left 5th, 6th, and 7th ribs, titanium bars, titanium clips were applied into the area of the ribs; pectoralis major and rectus abdominis advancement flap were replaced under the bar, than polypropylene mesh was placed over the bars and the reconstruction was completed with closing of skin. Patient was discharged on the 6th day, after uneventful postoperative follow-up. The pathologic report revealed low grade chondrosarcoma. He had no complaint of the chest at his 5th month outpatient control

## Discussion/Conclusion

For chondrosarcoma with wide resection in the treatment 97% 5-year survival is reported. Reconstruction with Titanium bar, polypropylene mesh, and chest wall muscle advancement flap has excellent stability, flexibility, and rigidity and permits a rapid return to baseline pulmonary mechanics.

## Consent

Written informed consent was obtained from the patient for publication of this abstract and any accompanying images. A copy of the written consent is available for review by the Editor of this journal

